# MUC1 expression in primary and metastatic pancreatic cancer cells for *in vitro* treatment by ^213^Bi-C595 radioimmunoconjugate

**DOI:** 10.1038/sj.bjc.6602232

**Published:** 2004-12-21

**Authors:** C F Qu, Y Li, Y J Song, S M A Rizvi, C Raja, D Zhang, J Samra, R Smith, A C Perkins, C Apostolidis, B J Allen

**Affiliations:** 1 Centre for Experimental Radiation Oncology, Cancer Care Centre, St George Hospital, Gray St Kogarah, NSW 2217, Australia; 2 University of New South Wales, NSW 2052, Australia; 3 Department of Surgery, Royal North Shore Hospital, NSW 2065, Australia; 4 University of Sydney, NSW 2006, Australia; 5 Department of Medical Physics, Medical School, Queen's Medical Centre, Nottingham, NG7 2UH, UK; 6 Institute for Transuranium Elements, Postfach 2340, D-76125 Karlsruhe, Germany

**Keywords:** MUC-1 mucin, pancreatic tumours, pancreatic cancer cell lines, C595 monoclonal antibody, *α*-particle emitter ^213^Bi, radioimmunoconjugate

## Abstract

Control of micrometastatic pancreatic cancer remains a major objective in pancreatic cancer treatment. The overexpression of MUC1 mucin plays an important role in cancer metastasis. The aim of this study was to detect the expression of MUC1 in human primary tumour tissues and three pancreatic cancer cell lines (CAPAN-1, CFPAC-1 and PANC-1), and target MUC1-positive cancer cells *in vitro* using ^213^Bi-C595 alpha-immunoconjugate (AIC). The expression of MUC1 on pancreatic tumour tissues and cancer cell lines was performed by immunohistochemistry and further confirmed by confocal microscope and flow cytometry analysis on the cell surface. Cytotoxicity of ^213^Bi-C595 was tested by MTS assay. Apoptosis was documented using TUNEL assay. Overexpression of MUC1 was found in ∼90% of tested tumour samples and the three pancreatic cancer cell lines. ^213^Bi-C595 is specifically cytotoxic to pancreatic cancer cells in a concentration-dependent fashion. These results suggest that overexpression of MUC1 in pancreatic cancer is a useful target, and that the novel ^213^Bi-C595 AIC selectively targets pancreatic cancer cells *in vitro*. ^213^Bi-C595 may be a useful agent for the treatment of micrometastases or minimal residual disease (MRD) in pancreatic cancer patients with overexpression of MUC1 antigen.

Pancreatic cancer is one of the leading cause of cancer death in the US, where an estimated 29 200 new cases per year will be diagnosed, and 28 900 people will die from the disease (Landis *et al*, 1999). The 5-year survival rate is only 5.2% for those who had no cancer-directed treatment (Sener *et al*, 1999) and the median survival is ∼6 months. Although some improvements in surgical outcome occur in patients who also receive chemotherapy and/or radiotherapy, the impact on long-term survival has not changed in two decades (Sener *et al*, 1999). The major problem in the management of postsurgical cases is failure to control cancer micrometastases. Many carcinoma-associated markers have aberrant expression in cancer cells. A monoclonal antibody (MAb) with high specificity and affinity could be used for targeted therapy, and may serve to overcome this problem.

MUC1 is a well-documented example of a marker that influences pathophysiological behaviour. High molecular weight glycoproteins, described as mucins or mucin-like glycoproteins, are frequently found associated with breast carcinoma and other epithelial cell adenocarcinomas (Zotter *et al*, 1988). Cancer-associated MUC1 is structurally different from normal MUC1 in that the former has shorter and less dense *O*-glycan chains, exposing novel regions of the protein core. The large extracellular domain is dominated by a heavily *O*-glycosylated region consisting of a variable number of 20 amino-acid tandem repeats (VNTRs) (Gendler *et al*, 1988). The number of these VNTR sequences is subject to genetic polymorphism, varying between 30 and over 100. MUC1 function involves mediating cellular transformation in integrating the growth factor receptor and Wnt signalling pathways (Ren *et al*, 2002). MUC1 expression causes anchorage-independent growth and tumour formation, and is a useful marker for the prognosis of the patients with carcinoma. Patients with MUC1 expression in the carcinoma show significantly lower survival rates than those without MUC1 expression (Kitamura *et al*, 1996; Utsunomiya *et al*, 1998).

MAb C595 (also known as NCRC48) is reactive with the protein core of MUC1 mucin. The target epitope of the MAb C595 is the tetrameric motif Arg-Pro-Ala-Pro that is repeated many times within the MUC1 protein core (Gendler *et al*, 1988; Price *et al*, 1990). The reactivity of the MAb C595 with synthetic peptides containing this motif permits efficient antibody purification using peptide-epitope affinity chromatography, which, unlike other methodologies, enables exclusion recovery of functionally active antibody.

MAbs are being realised for their potential in anticancer therapeutics. It was reported that the most widely explored strategy for enhancing the efficacy of antitumour antibodies is direct arming by chelator linkage to toxins or radionuclides (Farah *et al*, 1998). However, the evaluation of specific cancer MAbs remains a major task for antibody-based therapies. MUC1 may provide a good basis for targeting pancreatic cancer cells in transit or in preangiogenic cancer cell clusters. MAb C595 has been labelled with ^67^Cu and ^188^Re for radioimmunotherapy in bladder cancer (Hughes *et al*, 1997; 2000a; Murray *et al*, 2001).

Alpha-emitting radionuclides such as ^213^Bi emit *α* particles with energies of 4–8 MeV, which are up to an order of magnitude greater than most *β* rays. Yet, their ranges are two orders of magnitude less as *α* particles have a linear energy transfer (LET) that is about 100 times greater (Allen, 1999). Since *α* particles, by comparison with *β* particles, have a much shorter path length as well as a much higher LET, they are significantly more selective and potent in killing targeted cells (McDevitt and Scheinberg, 2002). Owing to the short path length, little collateral damage may be inflicted upon nontarget cells, while a single decay of an internalised *α*-emitter passing through the nucleus can be lethal (Sgouros *et al*, 1999; Jurcic *et al*, 2002). As a result, a much greater fraction of the total energy is deposited in cells with *α* particles and very few nuclear hits are required to kill a cell. Consequently, a 100-fold enhancement in radiation dose could be delivered to the nucleus of a cancer cell if a targeted vector is employed to take the *α*-radionuclide to that cancer cell.

In the present study, we have demonstrated moderate to strong MUC1 expression on the majority of human primary pancreatic tumours and pancreatic cancer cell lines using MAb C595. We also evaluated an anticancer effect of ^213^Bi-C595 against human pancreatic cells *in vitro*. We suggest that ^213^Bi-C595 may have the potential to target micrometastatic pancreatic cancer cells with MUC1 overexpression and represent a new therapeutic modality for the control of pancreatic cancer metastases.

## MATERIALS AND METHODS

### Monoclonal antibodies

C595 MAb was obtained from Nottingham University, UK. A nonspecific control IgG1 MAb (know as A2, offered MOPC) was provided by Professor A Collins (University of New South Wales, Australia). Rabbit anti-mouse IgG conjugated to HRP and mouse IgG1 negative control MAb were purchased from Dakopatts (Glostrup, Denmark). Goat anti-mouse-fluorescein isothiocyanate (FITC) MAb was purchased from Silenus (Sydney, NSW, Australia). Alexa Fluor 488 goat anti-mouse IgG MAb was purchased from Molecular Probes (Eugene, OR, USA).

### Human pancreatic tumour tissues

Following institutional approval from the Northern Sydney Health Human Research Ethics Committee, informed consent was obtained from 53 patients (male: 31, female: 22; age range 41–81years, average 68.7 years) undergoing pancreatic resection for a pancreatic mass at Royal North Shore Hospital, NSW, Australia. Tumour sections included normal pancreatic tissue. Slides from paraffin sections were prepared for H&E staining and immunostaining.

### Immunohistochemistry

MUC1 expression was detected by immunohistochemistry. Briefly, paraffin-embedded tissues were cut at 5 *μ*m sections, mounted on gelatine-coated glass slides and then incubated for 20 min at 60°C. The slides were deparaffinised in xylene, followed by a graded series of alcohols (100, 95 and 75%) and rehydrated in Tris-buffer saline (TBS, PH 7.5). The following steps were performed at room temperature (RT). The primary C595 MAb (13.0 *μ*g ml^−1^) was incubated for 1 h. After washing with TBS, slides were incubated with HRP-conjugated rabbit anti-mouse IgG (6.5 *μ*g ml^−1^) for 45 min and then washed with TBS two times, and developed with diamino benzoate (DAB) substrate solution for 5–10 min. The criteria for assessment are to combine staining intensity with percentage of positive cancer cells as follows: −, <25%; + (weak), 25–50%; ++ (moderate), 50–75%; +++ (strong), >75% of the neoplastic cells stained. An average of the grades for two independent observers is taken.

### Cell culture

The human pancreatic cell lines (CAPAN-1, CFPAC-1 and PANC-1) were purchased from American Type Culture Collection (ATCC, Rockville, MD, USA). CAPAN-1 and CFPAC-1 cells are cultured in Iscove's modified Dulbecco's medium supplemented with 20 and 10% (v v^−1^) heated-inactivated fetal bovine serum (FBS), 50 U ml^−1^ penicillin and 50 U ml^−1^ streptomycin, respectively. PANC-1 cells are cultured in Dulbecco's modified eagle's medium supplemented with 10% FBS, 50 U ml^−1^ penicillin and 50 U ml^−1^ streptomycin. All tissue culture reagents were supplied by Life Technologies Inc. (Grand Island, NY, USA), unless otherwise stated. Three cell lines were maintained in a humidified incubator at 37°C and 5% CO_2_ and detached with Dulbecco's phosphate-buffer saline (DPBS)/trypsin (0.25%)/ethylene diammine tetraacetic acid (EDTA) (0.05%) at 37°C for 5–10 min.

### Immunocytochemistry

An indirect conjugated peroxidase method was used to detect the expression of MUC1 on CAPAN-1, CFPAC-1 and PANC-1 pancreatic cancer cell lines as described previously (Li *et al*, 2002).

### Confocal microscope for MUC1 expression

1 × 10^5^ cultured cells including CAPAN-1, CFPAC-1 and PANC-1 were grown on coverslips. After washing with PBS, the cells were fixed on coverslips in ice-cold acetone for 10 min. C595 MAb (13.0 *μ*g ml^−1^) was incubated for 12 h at 4°C or for 2 h at RT on a shaking table and rinsed with PBS. Thereafter, a goat-anti-mouse-conjugated Alexa 488 green antibody (1 : 100 dilution) was added for 1 h at RT, rinsed in PBS for 10–15 min. The slides were mounted with coverslips using glycerol (Sigma-Aldrich Pty, Limited, Castle Hill, NSW, Australia). Examination was performed with Confocal Microscope (FV300/FV500 Olympus, Japan).

### Flow cytometry

Indirect immunofluorescence imaging counter was performed to detect cell-surface expression of MUC1 in CAPAN-1, CFPAC-1 and PANC-1 pancreatic cancer cell lines as described previously (Li *et al*, 2002).

### Preparation of *α* conjugates

^213^Bi has a short half-life (*t*_1/2_=46 min), short range (80 *μ*m) and emits an *α* particle with energy of 8 MeV. ^213^Bi was eluted from the ^225^Ac/^213^Bi column, which was supplied by the Institute for Transuranium Elements (ITU), Germany, with 250 *μ*l of freshly prepared 0.15 M hydriodic acid (HI) as the (BiI_5_)^2−^ anion species, neutralised to pH 4–4.5 with the addition of 3 Mammonium acetate and immediately used to radiolabel the MAb construct (Boll *et al*, 1997). A time of 2–3 h was allowed for ^213^Bi to grow back in the generator for the next elution. C595 and A_2_ MAbs were conjugated with the chelator, cyclic diethylenetriaminepentacetic acid anhydride (cDTPA) (Sigma-Aldrich Pty, Limited, Castle Hill, NSW, Australia), using published methods (Abbas Rizvi *et al*, 2000). The conjugated C595 and A2 were measured by plate reader at 280 nm wavelength using ProMax software (Bio-TEC Instruments Inc., Winooski, Vermont, USA), and purified on a PD-10 column (Amersham Biosciences Pty Limited, Castle Hill, NSW, Australia). Alpha-immunoconjugates of each antibody were obtained by labelling with free ^213^Bi for 20 min at RT. The radiolabelling efficiency was determined by instant thin layer chromatography (ITLC) using a Gelman paper (Gelman Science Inc., Ann Arbor, MI, USA). Briefly, a 10 *μ*l aliquot of the final reaction mixture was applied to Gelman paper. The paper strips were developed using 0.5 M sodium acetate (pH 5.5) as the solvent. The paper strips were cut into four sections and the gamma emissions from the radioisotope in each section were counted using a 340–540 keV window. The radiolabelled protein stayed at the origin section, while free radioisotope moved with the solvent front section. The labelling efficiency of AICs was ∼93%. Specific activity was in the range 1–1.5 *μ*Ci *μ*g^−1^.

### *In vitro* cell cytotoxicity

An MTS assay was used to test cell cytotoxicity after treatment. Cultured CAPAN-1, CFPAC-1 and PANC-1 pancreatic cancer cells were washed twice with DPBS and seeded into 96-well flat-bottomed plates at a density of 2 × 10^4^ cells in 100 *μ*l in RPMI-1640 medium plus 5% FBS. The activity of ^213^Bi-C595 (test) and ^213^Bi-A_2_ (control) preparations was measured using a radioisotope calibrator (Biodex Medical System ATOMLAB 200, Shirley, NY, USA) and neutralised to pH 7.0 via the addition of 10% (v v^−1^) 1 M NaHCO_3_ (pH 9.0). After this, six serial activities of ^213^Bi-C595 AIC (1∼10 *μ*Ci) were added to each well in triplicate. Controls included nonspecific ^213^Bi-A2 (10 *μ*Ci), cDTPA-C595, C595 MAb alone or cell alone (no treatment) were also performed in triplicate in the same 96-well plate for each experiment. The plates were then incubated o/n in a 5% CO_2_ atmosphere at 37°C. The cells were then washed and incubated with 100 *μ*l phenol-red free medium (without FBS) containing 20 *μ*l of the cellTiter 96 Aqueous One Solution reagents (Promega, Madison, WI, USA). After a 3 h incubation in a 5% CO_2_ atmosphere at 37°C, the reaction was stopped by the addition of 10% SDS, and the absorbance in each well was recorded at 490 nm using a SPECTRO max plate reader (BIO-RAD, Hercules, CA, USA). The absorbance reflects the number of surviving cells. Blanks were subtracted from all data and analysed using Prism software (GraphPad Software Inc., USA).

### TUNEL assay

A TUNEL assay was performed to investigate whether the lethal pathway is through apoptosis after treatment with ^213^Bi-C595. Briefly, CAPAN-1, CFPAC-1 and PANC-1 pancreatic cancer cells were cultured in six-well plates and incubated in medium as described above. The cultured cells were treated with ^213^Bi-C595 AIC in a concentration of 1 *μ*Ci 10^4^ cells^−1^ or with nonspecific control AIC ^213^Bi-A2 at the same concentration, and media alone at 37°C for 4, 8, 12, 24, 48 and 72 h. After treatment, the cells were washed with DPBS, harvested by centrifugation of the washing DPBS and slides were made by cytospin. Apoptosis cells were detected using the TUNEL method (Gavrieli *et al*, 1992) with TdT-fragEL™ *in situ* apoptotic detection kit according to the manufacturer's instructions (Oncogene Research Products, Boston, MA, USA). Specificity of TUNEL reactivity was confirmed by undertaking in parallel appropriate negative (omitting TdT from the labelling mix) and positive (treated HL-60 slides) controls. Cells with three or more nuclear chromatin fragments were considered as positive apoptosis. The labelled cells were examined using a Leica light microscope (Leica microscope, Nussloch, Germany) at × 40 magnifications. The results were expressed as a percentage of total cells staining positive for apoptosis.

## RESULTS

### Expression of MUC1 on human pancreatic cancer tissues

Immunoreactivity identified in pancreatic cancer tissues using paraffin section stained with MAb C595 is summarised in Table 1

**Table 1:** Intensity of immunohistochemical staining of pancreatic cancer tissues and normal pancreas tissues

	**Tissue staining with C595 MAb**			
	**+++**	**++**	**+**	**-**
Tumour	28	15	5	5
Normal pancreas	0	0	3	50

Cancer and normal tissue sections were collected from 53 pancreatic cancer patients. −=negative staining; +=weak staining; ++=moderate staining; +++=strong staining.

**Figure 1:**
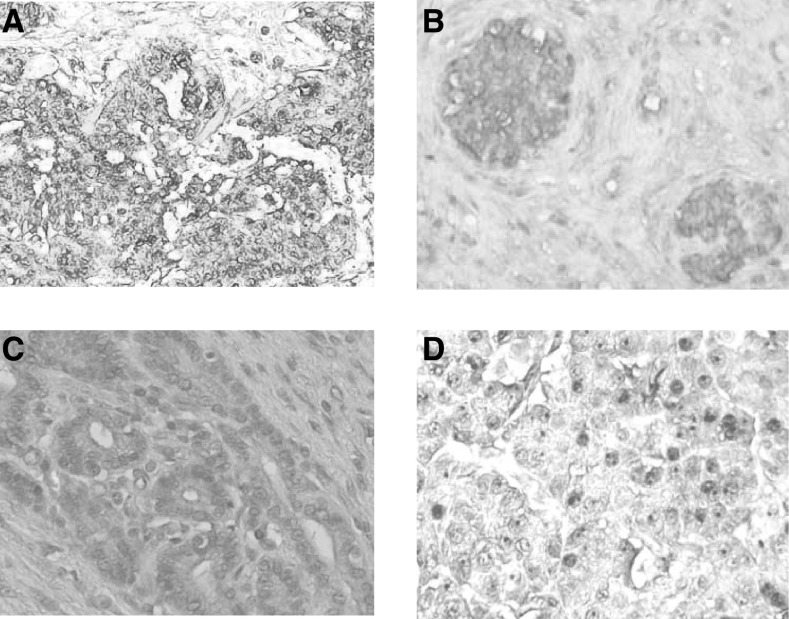
Expression of MUC1 in pancreatic cancer tissues. MUC1 expression was assessed by immunohistochemistry. Representative pictures are shown for immmunostaining with MAbs C595 (test) and A2 (control). High expression of MUC1 was found in patient 1 staining with MAb C595 (**A**) and moderate expression was found in patient 2 staining with MAb C595 (**B**), while no MUC1 expression was found in patient staining with MAb A2 (**C**) or in normal pancreas (**D**).

. Typical staining results are shown in Figure 1

. Strong MUC1 expression was found in 28 out of 53 (53%) tested samples (Figure 1A, patient 1). Moderate MUC-1 expression was found in 15 out of 53 (28.4%) (Figure 1B, patient 2), and weak expression in five out of 53 (9.4%) samples. No significant expression of MUC1 was found in five out of 53 (9.4%) samples. The control A2 MAb was negative to pancreatic cancer section (Figure 1C). Normal tissues were not detected with immunoreactivity in 50 out of 53 (94.6%) (Figure 1D). Only three samples with weak immunoreactivity were detected in the normal part of the pancreatic gland.

### Expression of MUC1 in pancreatic cancer cell lines

The immunoreactivity of the three pancreatic cancer cell lines to MAb C595 is summarised in Table 2

**Table 2:** Summary of immunocytochemistry, flow cytometry, confocal microscope and cytotoxicity results

	**MUC1**				
		**Flow cytometry^a^**			
	**Immunocytochemistry**	**Gated %**	**Median value**	**Confocal microscope**	**Cytotoxicity *D*_0_ value**
CAPAN-1	++	95	40	++	4.5
CFPAC-1	+++	99.5	500	+++	3.8
PANC-1	+++	98.5	80	+++	4.3

−=negative staining; +=weak staining; ++=moderate staining; +++=strong staining.

a positive cells percentage.

**Figure 2:**
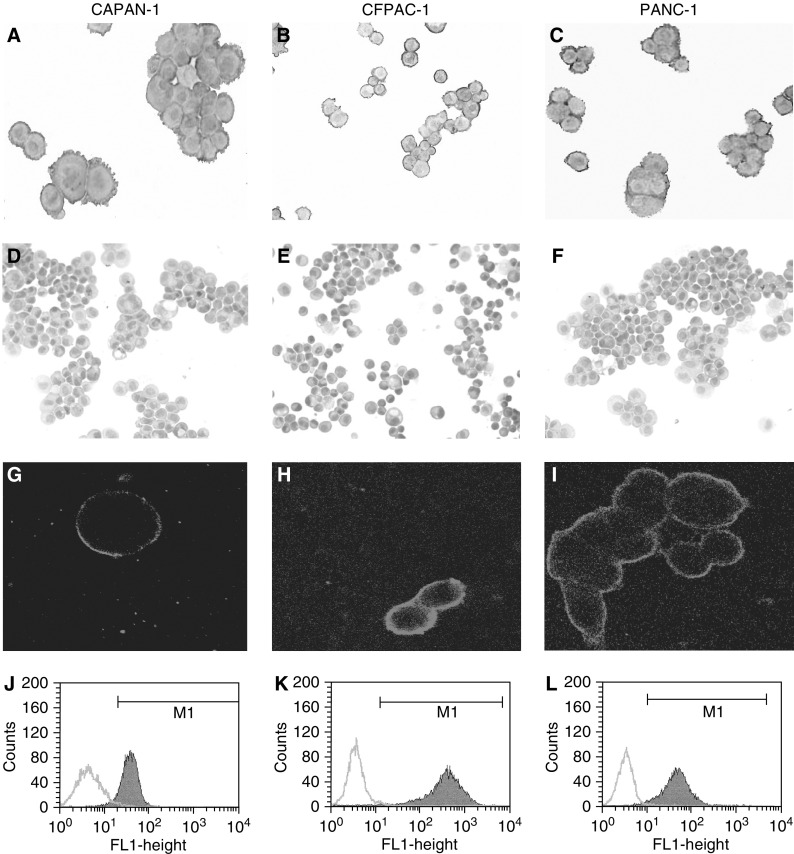
Expression of MUC1 in pancreatic cancer cell lines. Representative pictures are shown for immmunostaining with MAbs C595 (test) and A2 (control). MAb C595 was strongly positive in three cell lines (**A–C**), while MAb A2 was negative in three cancer cell lines (**D–F**). Brown staining indicates positive cells. The MUC1 cell surface expression in three cancer cell lines was confirmed by Confocal Microscope (**G–I**). Green staining indicates positive cells. Expression of MUC1 in three viable pancreatic cancer cell lines was assessed by FACA analysis (**J–L**). Data are presented as histograms, using a mouse IgG1-negative control to determine background fluorescence and to set the marker (M1). All of the photographs are at × 200 magnification.

. Immunocytochemical staining of CAPAN-1, CFPAC-1 and PANC-1 cell lines was positive for MAb C595 (Figure 2A, B and C

), but negative for control MAb A2 (Figure 2D, E and F) or omitted primary MAb (data not shown). These results indicate that high expression of target antigens (MUC1) was found in metastatic and primary pancreatic cancer cell lines.

Immunocytochemical staining appeared to localise to the cell membranes and Confocal Microscopy confirmed this. The Confocal results indicate that surface expression of MUC1 was found in CAPAN-1 (Figure 2G), CFPAC-1 (Figure 2H) and PANC-1 (Figure 2I) using MAb C595, but not for MAb A2 (data not shown). Flow cytometry analysis also demonstrated distinct differences in the patterns of reactivity of the MAb C595 with three cancer cell lines compared with control MAb as shown by the representative histograms. There were positive shifts (increases in the intensity of fluorescence) in the CAPAN-1 (Figure 2J), CFPAC-1 (Figure 2K) and PANC-1 (Figure 2L) cells stained with MAb C595. These results indicate that the target antigen (MUC1), recognised by MAb C595, are on the cell surface of viable cells, and confirm our immunocytochemical observations.

### ^213^Bi-C595 inhibits pancreatic cancer cells proliferation *in vitro*

**Figure 3:**
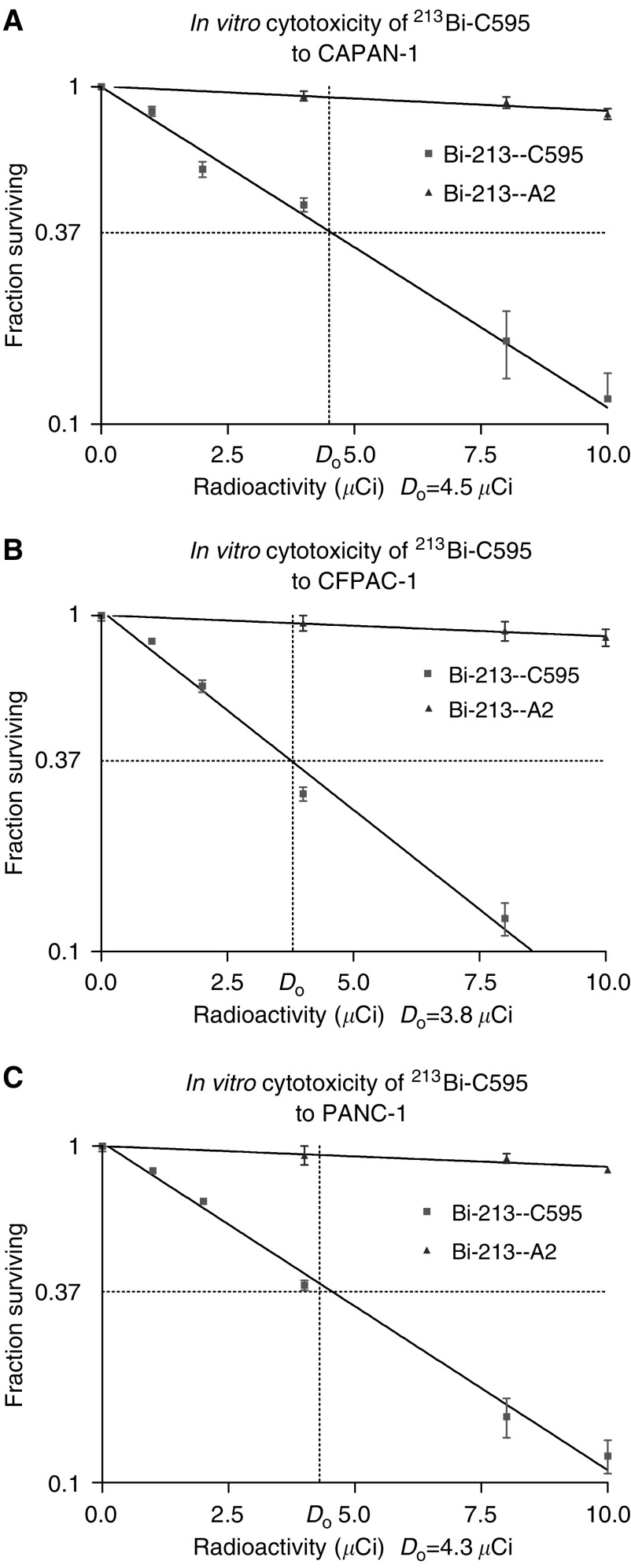
Representative cytotoxicity study of CAPAN-1 (**A**), CFPAC-1 (**B**) and PANC-1 (**C**) cells following treatment for 24 h with ^213^Bi-C595. Cells were treated with varying concentrations of ^213^Bi-C595 or nonspecific control ^213^Bi-A2, incubated overnight and cell survival was measured by MTS assay at 24 h and expressed as a percentage of cell survival of control cells. Results are expressed as a mean percent s.d. of control plates containing nonspecific *α* conjugates. Each experiment was performed in triplicate, and each point represents the mean of three experiments.

Varying concentrations of ^213^Bi-C595 were added to the cultured cell lines for 24 h, and their effect on cell growth was assessed using MTS assays in triplicate. *D*_0_ (37% cell survival) values of ^213^Bi-C595 are at 3.8, 4.5 and 4.3 *μ*Ci in 300 *μ*l for three cell lines (Figure 3

), whereas the *D*_0_ values of ^213^Bi-A2 are at 60–69 *μ*Ci, 13–18-fold, respectively. ^213^Bi-C595 AIC inhibits the growth of the three pancreatic cancer cell lines *in vitro* in a concentration-dependent fashion. At the maximum dose of 10 *μ*Ci, cell survival when treated with ^213^Bi-C595 was reduced to 5–10%, while those treated with nonspecific ^213^Bi-A2 AIC was 90–95%, and cDTPA-C595 or C595 MAb alone were more than 95%. The higher the overexpression of MUC-1, the lower the *D*_0_ value (See Table 2).

### ^213^Bi-C595 induces apoptosis

**Figure 4:**
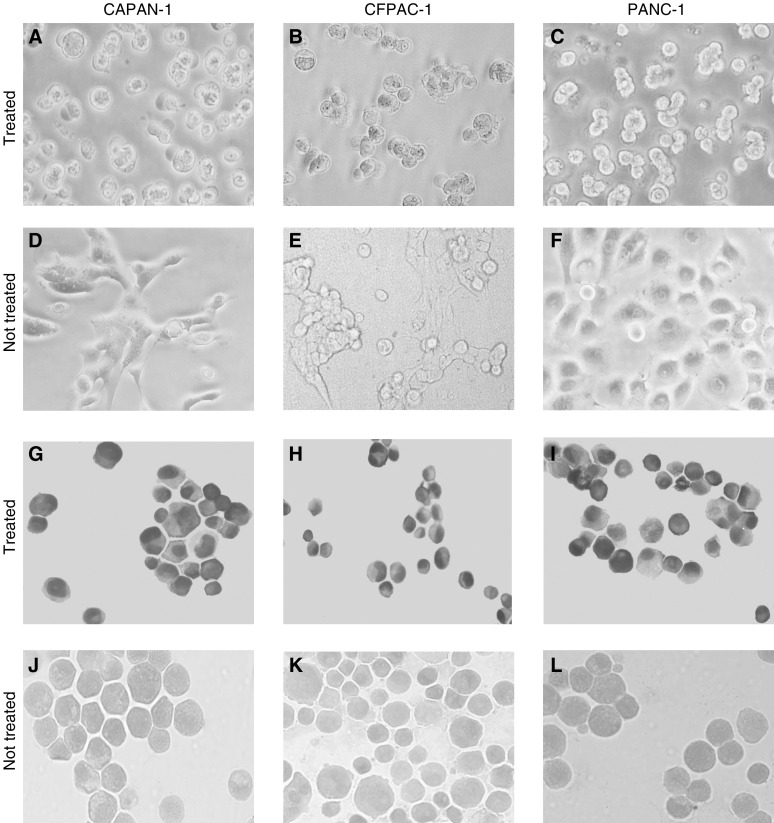
Morphological changes in three cell lines incubated with ^213^Bi-C595 (**A–C**) for 24 hours. (**D–F**) Matched, untreated cells are shown. TUNEL assays of CAPAN-1, CFPAC-1 and PANC-1 cells are shown in (**G–I**), and control in (**J–L**). Typical apoptotic cells with condensed or fragmented nuclei are observed in treated cell lines, while control cells show normal shapes. All of the photographs are at × 200 magnification.

After treatment with ^213^Bi-C595, the treated cells in the 96-well plates showed typical apoptotic morphology, that is, cells became rounded, shrunken and detached, whereas untreated controls and cells treated with the nonspecific control ^213^Bi-A2 did not exhibit apoptotic morphology. Representative morphological changes are shown in Figure 4A–F

**Figure 5:**
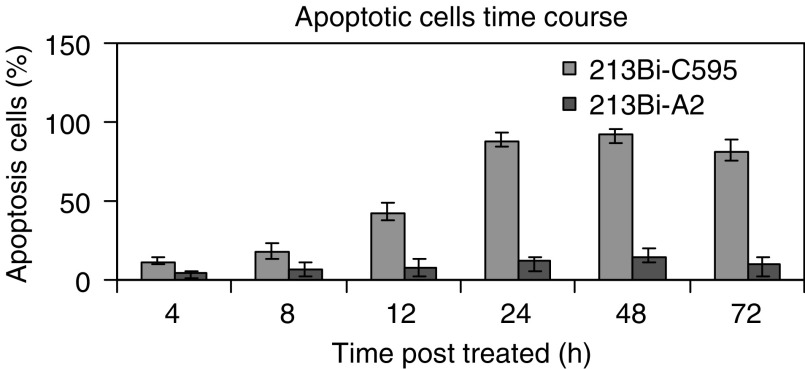
Observation of apoptosis in time course after treatment with ^213^Bi-C595 and ^213^Bi-A2 AICs. Blue and red columns represent ^213^Bi-C595 and ^213^Bi-A2, respectively. The PANC-1 cells were treated with ^213^Bi-C595 and ^213^Bi-A2 with concentrations of 10 *μ*Ci 10^5^cells^−1^, incubated at 4, 8, 12, 24, 48 and 72 h, tested by TUNEL assay, and expressed as a percentage of TUNEL-positive cells in total cells. Each experiment was performed in triplicate, and each point represents the mean of three experiments.

. The exposed 3′−OH ends of DNA fragments generated by apoptotic DNA cleavage were detected by TUNEL assay, in which the nonapoptotic cells stained green, while apoptotic cells stained brown. A representative experiment is shown in Figure 4G–L. After treatment, ^213^Bi-C595-treated cells showed typical apoptotic morphology, whereas cells treated with ^213^Bi-A2 did not. The results are summarised in Figure 4. ^213^Bi-C595 causes morphological changes of treated cancer cells and induces apoptosis. The percentages of apoptotic cells are 11, 18, 42, 87, 92 and 81% at 4, 8, 12, 24, 48 and 72 h after treatment with 10 *μ*Ci 10^5^ cells^−1^, respectively (Figure 5

). However, the percentage of apoptotic cells for nonspecific control groups remains low (<15%).

## DISCUSSION

Monoclonal antibody-based therapeutics is an important option to control metastases and improve survival rate of pancreatic cancer. These strategies include MAb combination with cytotoxic drugs, conjugation with radionuclides or immunological effector cells. High specification and affinity to targeted cancer tissue are essential for the selection of targeted antigens and targeting vectors.

In the present study, we first demonstrated the expression of the MUC1 antigen on human pancreatic cancer specimens and human pancreatic cancer cell lines using MAb C595. MUC1 is a mucin, high MW glycoprotein. The biological function of MUC1 may be in part due to its large size and the extended rigid structure. The expression of MUC1 in tumours may function as an antiadhesion molecule, which inhibits cell–cell adhesion, inducing a release of cells from tumour nests and causing micrometastasis (Feng *et al*, 2002). Overexpression of MUC1 in cancer cells may effect efficient lysis by cytotoxic lymphocytes and therefore contribute to escape from immune surveillance (van de Wiel-van Kemenade *et al*, 1993). It has been proposed that enhanced levels of MUC1 expression by cancer cells may mask extracellular domains from immune surveillance, confer a survival advantage on malignant cells and play an important role in the ability of tumours to invade and metastasise (Mommers *et al*, 1999; Hughes *et al*, 2000b). It was also reported that increased MUC-1 expression correlates with the stage and grade of pancreatic cancer. MUC1 upregulation was significantly correlated to the depth of invasion, lymph node metastasis and peritoneal dissemination (Satoh *et al*, 2000). In this study, we found that over 90% of primary pancreatic tumours expressed MUC1, while the majority of normal pancreases did not. This finding is significant because this may provide a useful target for TAT in postsurgical MRD while sparing the normal pancreas.

Using the MAb C595, we found MUC1 expression on the surface of CAPAN-1, CFPAC-1 and PANC-1 cancer cells by flow cytometric analysis and confocal microscopy. CAPAN-1 and CFPAC-1 are metastatic cancer cell lines, while PANC-1 is a primary cancer cell line. Overexpression of MUC1 was found in all three cell lines, suggesting that this overexpression may involve pancreatic cancer metastases and that cancer clones that escape from primary tumours do not lose the MUC1 antigen. This means that targeting by systemic radiolabelled MAb C595 may be possible. Our results clearly indicated that MUC1 is an effective tumour surface marker for targeting pancreatic carcinoma. The overexpression of MUC1 can provide a good target for further investigating *in vitro* efficacy.

Cytotoxicity of ^213^Bi-C595 to three pancreatic cancer cells proved to be specific and concentration-dependent. Three cell lines demonstrated a similar pattern of cell killing because the expression of MUC1 on three cell lines is similar (see Table 2). These results suggest ^213^Bi-C595 can target and kill 63% of pancreatic cancer cells (monolayer) *in vitro* using a low dose (3.8∼4.5 *μ*Ci). Using 10 *μ*Ci of ^213^Bi-C595, only <10% cancer cells can survive after treatment, while using the same activity of ^213^Bi-A2, >90% cancer cells can survive. There was a 13∼18-fold difference in the 37% survival (*D*_0_) values *in vitro* for the test ^213^Bi-C595 compared with a nonspecific control ^213^Bi-A2. There was no cell killing for cDTPA-C595 and C595 groups. These results suggest that only specific ^213^Bi-C595 can effectively target MUC1-positive cancer cells.

The exact mechanism of cell killing using ^213^Bi-C595 is still not clear. One explanation is that after binding the surface MUC1 antigen, ^213^Bi-C595 may form ^213^Bi-C595–MUC1 complexes at the cell membrane, emitting *α* particles that kill pancreatic cancer cells by causing double-DNA-strand breaks. Another possibility is that the surface-bound ^213^Bi-C595–MUC1 complexes may be internalised, resulting in increased cell killing efficiency. Clearly, many factors including antigen affinity and antigen density will also play an important role in the killing of targeted antigen-positive cells, since those with high density will attract more AIC and those with higher antigen affinity may ‘hold on’ to increased levels of radioactivity for a longer time period. The relative importance of these factors (antigen density, antigen affinity and internalisation) in the killing process has not yet been determined.

The most effective radiation treatments are those that not only hit the intended target but also cause the greatest amount of lethal or nonrepairable damage to DNA. Therefore, *α* particles are most effective in this respect (Hall, 1994). A large number of *in vitro* and *in vivo* experiments with *α*-immunotherapy have shown dramatic superiority over *β*-immunotherapy as only a few *α* hits of the nucleus are needed to kill cells (Scheinberg *et al*, 1982; Vaughan *et al*, 1982; Allen, 1999; Kennel *et al*, 1999; McDevitt *et al*, 2000). Using electron micrographs in studies of murine lymphoma, Macklis *et al* (1992) have demonstrated bizarre blebbing patterns, condensation of chromosomal material and internucleosomal DNA fragmentation patterns characteristic of programmed cell death (apoptosis) after treatment with AIC, and suggested that *α*-particles may kill cells by apoptotic mechanisms. In this study, we found typical morphologic changes and a high percentage of TUNEL-positive cells in three pancreatic cancer cell lines after treatment using ^213^Bi-C595 *in vitro*. These data suggest that the lethal pathway for the three cell lines *in vitro* after TAT involves apoptosis.

## CONCLUSION

We have demonstrated moderate to strong MUC1 expression on the majority of human primary tumours and pancreatic cancer cell lines using MAb C595. MUC1 is therefore an ideal targeted antigen for targeted *α* therapy using ^213^Bi-C595. This AIC can target and selectively kill pancreatic cancer cells *in vitro*. The lethal pathway involves apoptosis. The specific cytotoxic effects obtained in this *in vitro* study suggest this AIC may target and kill the majority of human primary and metastatic pancreatic cancer cells harbouring moderate to strong MUC1 overexpression.
